# Depletion of m^6^A-RNA in *Escherichia coli* reduces the infectious potential of T5 bacteriophage

**DOI:** 10.1128/spectrum.01124-24

**Published:** 2024-10-18

**Authors:** Bibakhya Saikia, Sebastian Riquelme-Barrios, Thomas Carell, Sophie Brameyer, Kirsten Jung

**Affiliations:** 1 Faculty of Biology, Microbiology, Ludwig-Maximilians-Universität München, Martinsried, Germany; 2 Department for Chemistry, Institute for Chemical Epigenetics (ICE-M), Ludwig-Maximilians-Universität, München, Germany; Lerner Research Institute, Cleveland, Ohio, USA

**Keywords:** epitranscriptome, RNA modification, phage replication, *Vibrio campbellii*, lysogeny, Virtus

## Abstract

*N*^6^-Methyladenosine (m^6^A) is the most abundant internal modification of mRNA in eukaryotes that plays, among other mechanisms, an essential role in virus replication. However, the understanding of m^6^A-RNA modification in prokaryotes, especially in relation to phage replication, is limited. To address this knowledge gap, we investigated the effects of m^6^A-RNA modifications on phage replication in two model organisms: *Vibrio campbellii* BAA-1116 (previously *Vibrio harveyi* BB120) and *Escherichia coli* MG1655. An m^6^A-RNA-depleted *V. campbellii* mutant (Δ*rlmF*Δ*rlmJ*) did not differ from the wild type in the induction of lysogenic phages or in susceptibility to the lytic Virtus phage. In contrast, the infection potential of the T5 phage, but not that of other T phages or the lambda phage, was reduced in an m^6^A-RNA-depleted *E. coli* mutant (Δ*rlmF*Δ*rlmJ*) compared to the wild type. This was shown by a lower plaquing efficiency and a higher percentage of surviving cells. There were no differences in the T5 phage adsorption rate, but the mutant exhibited a 5-min delay in the rise period during the one-step growth curve. This is the first report demonstrating that *E. coli* cells with lower m^6^A-RNA levels have a higher chance of surviving T5 phage infection.

**IMPORTANCE:**

The importance of RNA modifications has been thoroughly studied in the context of eukaryotic viral infections. However, their role in bacterial hosts during phage infections is largely unexplored. Our research delves into this gap by investigating the effect of host *N*^6^-methyladenosine (m^6^A)-RNA modifications during phage infection. We found that an *Escherichia coli* mutant depleted of m^6^A-RNA is less susceptible to T5 infection than the wild type. This finding emphasizes the need to further investigate how RNA modifications affect the fine-tuned regulation of individual bacterial survival in the presence of phages to ensure population survival.

## INTRODUCTION

Chemical modifications of RNA molecules are reversible and dynamic and influence their fate and many cellular processes (1). Of the numerous RNA modifications discovered to date, the eukaryotic *N*^6^-methyladenosine (m^6^A) modification machinery and its presence in host and viral RNA are especially well studied and provide a potential target for antiviral therapeutics (1–7). For example, infection of human CD4 T cells with human immunodeficiency virus 1 leads to an increase in m^6^A modification levels in both host and viral transcripts (8). DNA viruses such as adenovirus, simian vacuolating virus 40 (SV40), and herpes simplex virus 1 have transcripts that are highly modified with m^6^A, which promotes their replication (9–11). Despite this extensive understanding of m^6^A-RNA modification in eukaryotes and eukaryotic viruses, the role of m^6^A-RNA modifications in bacteria, particularly in relation to bacteriophages (phages), remains largely unexplored.

Phages are prokaryotic viruses that infect bacteria and use them as hosts to propagate. They are ubiquitous and known to be highly abundant in various natural ecosystems such as soil and water, in addition to the human microbiome (12). Their varied and omnipresent lifecycles reflect their complex interactions with bacterial hosts, which influence their population, virulence, and evolution (13). These complex interactions have attracted considerable research interest related to the intricate fine-tuning between hosts and phages. For example, phage-encoded transfer RNAs (tRNAs) are modified like the corresponding host tRNAs, suggesting that they are processed by the same host enzymes (14). Similarly, a preprint is available that describes the bacterial toxin CmdT (part of the toxin–antitoxin–chaperone system), which acts as adenosine diphosphate (ADP)-ribosyltransferase and modifies phage mRNA to block protein translation (15). Thus, as with most eukaryotic viruses, RNA modifications in phages may be performed by host enzymes and may have pro- or antiviral functions.

Adenosine methylation at the nitrogen (m^6^A) is present in all types of *Escherichia coli* RNA (16); however, the methyltransferases are only well documented for ribosomal RNA (rRNA) and tRNA. In *E. coli*, 23S rRNA molecules are methylated at nucleotides A1618 and A2030 by RlmF and RlmJ, respectively (17, 18). In addition, the valine-specific tRNA is methylated at nucleotide A37 by TrmM (YfiC) (19). Notably, RlmF is a structural homolog of METTL16, a mRNA methyltransferase in eukaryotes (20). The overexpression of *rlmF* leads to a slight growth defect in *E. coli* (17), whereas the deletion of *rlmJ* leads to a higher growth rate under anaerobic conditions (18). Single deletions of either *rlmF* or *rlmJ* do not interfere with ribosome assembly or influence the growth rate in *E. coli* (21). RlmJ is conserved in the phylum Proteobacteria, whereas RlmF is predominantly found in the class *Gammaproteobacteria* (21). Consequently, RlmF, RlmJ, and TrmM are well conserved in the marine bacterium *Vibrio campbellii* BAA-1116 (previously known as *Vibrio harveyi* BB120), a model organism for quorum sensing (22). Our previous work with *V. campbellii* has shown the presence of four intact prophages in the *V. campbellii* genome, two of which can be activated under oxidative (mitomycin C) and heat stress (23).

To explore the potential role of m^6^A-RNA modification in lysogenic and lytic phages, we tested *V. campbellii* and *E. coli* m^6^A-RNA depletion mutants lacking both *rlmF* and *rlmJ,* which encode the major m^6^A rRNA methyltransferases. We started our study by the induction of lysogenic phages in *V. campbellii* and could show that m^6^A-RNA modification has no significant effect on prophage induction. We then searched for lytic phages that showed different infection efficiencies in the m^6^A-RNA-depleted mutants. We identified bacteriophage T5, whose infectious potential was reduced in the m^6^A-RNA-depleted *E. coli* mutant, resulting in higher host survival.

## RESULTS

### m^6^A-RNA depletion in *V. campbellii* does not affect prophage induction or susceptibility to a lytic phage

**Fig 1:**
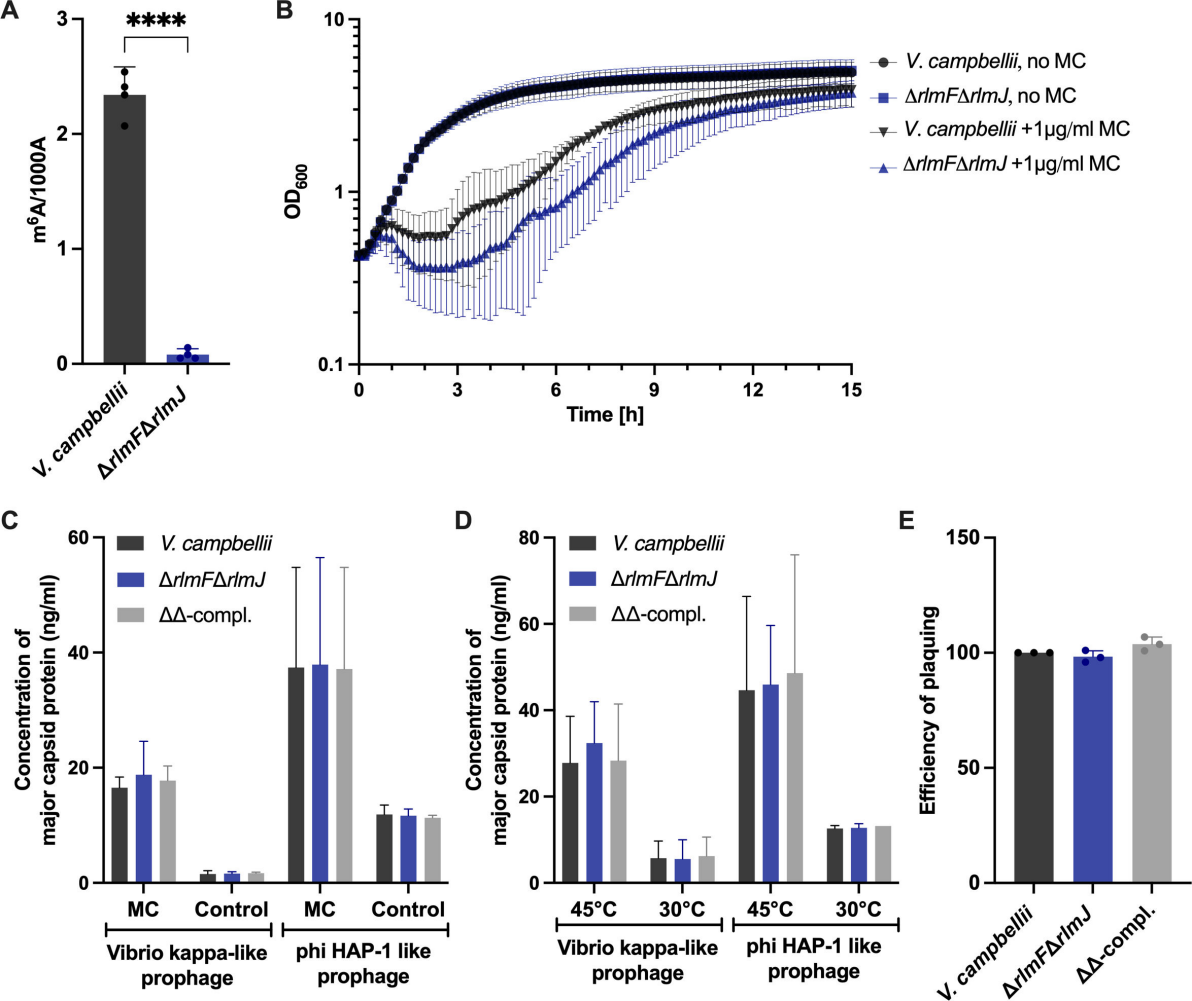
The effect of m^6^A-RNA depletion in *V. campbellii* ATCC BAA1116 on the induction of lysogenic phages and susceptibility to lytic Virtus phage. (**A**) Quantitative analysis of m^6^A related to 1000 A (adenosine) of isolated rRNA and mRNA fraction from *V. campbellii* and its Δ*rlmF*Δ*rlmJ* mutant. (**B**) The indicated strains were grown to the exponential growth phase and exposed to 1 µg/mL mitomycin C (MC) for 30 min. Afterward, cells were resuspended in a fresh medium, and optical density at 600 nm (OD_600_) was monitored over time. (**C and D**) Lysogenic phages were induced in the indicated strains with 1 µg/mL MC for 30 min (**C**) or heat at 45°C for 30 min (**D**). Culture supernatants were collected 2 hours after induction and subjected to indirect enzyme-linked immunosorbent assay (ELISA) to estimate the release of *Vibrio* kappa-like prophages and ΦHAP-1-like prophages. (**E**) The indicated strains were grown to the exponential growth phase and infected with phage Virtus. The graph shows the efficiency of plaquing (EOP), which is calculated by dividing the number of plaques obtained from the mutant by the number of plaques obtained from the wild type. The EOP of the wild type was set to 100. Data are presented as the mean ± standard deviation of at least three independent experiments. ^****^P < 0.0001 (Student *t*-test).

*V. campbellii* has four intact prophages in its genomes (23). Prophages are viral DNA segments that have integrated into the host genome (also called lysogen) and provide a competitive advantage to the host (24). In *V. campbellii*, ΦHAP-1-like prophage and *Vibrio* kappa-like prophage are triggered into their lytic cycle under oxidative or heat stress (23). Prophage induction triggers a transition from the lysogenic to the lytic cycle, resulting in the activation of dormant viral DNA and subsequent viral replication and host cell lysis. Knowing the effects of oxidative and heat stress on prophage induction in the wild type of *V. campbellii*, we wanted to determine whether the m^6^A-RNA-depleted mutant (Δ*rlmF*Δ*rlmJ*) shows a different response. We used liquid chromatography with tandem mass spectrometry (LC-MS/MS) to quantify the m^6^A content in the tRNA-depleted rRNA and mRNA samples of wild-type *V. campbellii* and found that the m^6^A/A ratio was approximately 0.2% (Fig. 1A). The deletion of *rlmF* and *rlmJ* in *V. campbellii,* which encode the two m^6^A-specific rRNA methyltransferases, resulted in a decrease of 96.5% in m^6^A levels of the tRNA-depleted mRNA and rRNA fraction (Fig. 1A).

First, we tested the role of m^6^A-RNA on lysogeny. We exposed the wild type and the ∆*rlmF*∆*rlmJ* mutant to mitomycin C and monitored the cell lysis caused by phage release over time. At 2.5 hours after exposure to mitomycin C, a greater extent of cell lysis was observed in the ∆*rlmF*∆*rlmJ* mutant compared to the wild type, but the effect was not statistically significant (Fig. 1B). In parallel, we raised primary antibodies against two major capsid proteins—one targeting the *Vibrio* kappa-like prophage (VIBHAR_05027) and the other targeting ΦHAP-1-like prophage (VIBHAR_01983)—to study their induction under oxidative or heat stress. An indirect enzyme-linked immunosorbent assay (ELISA) was employed to measure major capsid proteins in the supernatant to quantify the total phage particles released by the host. Since our phage lysate does not reinfect its host and transmission electron microscopy has confirmed the presence of intact phage particles (23), the indirect ELISA method is valuable for evaluating the phage load in our samples, even though it cannot directly assess phage infectivity.

Our results revealed that higher levels of capsid proteins were detected for the ΦHAP-1-like prophage than for the *Vibrio* kappa-like prophage, suggesting that the former was more strongly induced under oxidative or heat stress (Fig. 1C and D). However, no significant differences were observed between the wild type, the m^6^A-RNA-depleted mutant, or the complemented mutant (Fig. 1C and D). Furthermore, low levels of capsid proteins were observed in the untreated cells, likely due to the spontaneous induction of prophages (Fig. 1C and D).

We then tested whether the m^6^A-RNA modification has an effect during lytic phage infection (Fig. 1E). The availability of the vibriophage Virtus, which is capable of infecting a broad range of *Vibrio* spp. (including *V. campbellii*) (25), provided the unique opportunity to evaluate its infectivity for *V. campbellii* wild type and its Δ*rlmF*Δ*rlmJ* mutant. We tested the efficiency of plaquing by phage Virtus after the infection of exponentially grown *V. campbellii* wild type, its Δ*rlmF*Δ*rlmJ* mutant, and the complemented mutant (ΔΔ-compl.). However, infectivity by Virtus was comparable for all three strains (Fig. 1E). We also tested the efficiency of plaquing by phage Virtus after the infection of stationary phase cells of the *V. campbellii* strains but could not detect any difference (data not shown).

In summary, the m^6^A-RNA modification had no effect on prophage induction or replication of the lytic Virtus phage in *V. campbellii*.

### m^6^A-RNA depletion in *E. coli* affects bacteriophage T5 infection

Since further exploration of the role of m^6^A-RNA modification in *V. campbellii* has been hampered by the lack of easily available lytic phages, we proceeded with *E. coli*, which, although containing cryptic prophages in its genome (26), offers a diverse array of well-characterized lytic phages (27). An m^6^A-RNA-depleted mutant (∆*rlmF*∆*rlmJ*) *E. coli* MG1655, which has a reduction in the m^6^A-RNA level comparable to that of *V. campbellii* (Fig. 1A), and a complemented mutant were already available (28).

**Fig 2:**
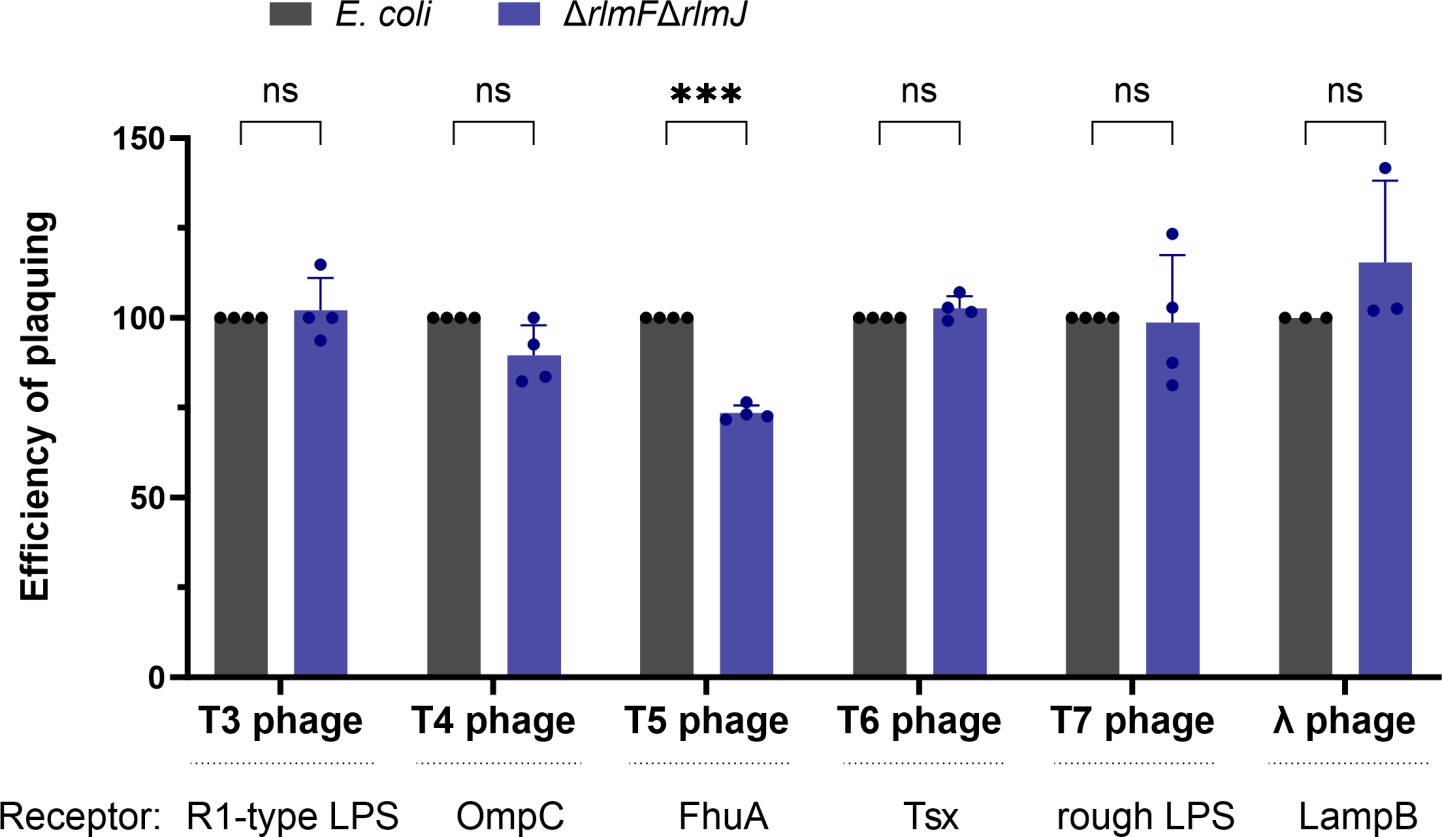
Depletion of m^6^A-RNA in *E. coli* MG1655 and its effect on infection by different lytic phages. *E. coli* wild type and the ∆*rlmF*∆*rlmJ* mutant were grown to an OD_600_ = 0.5 before infection with the different phages. The EOP was calculated by dividing the number of plaque-forming units (PFU) of the ∆*rlmF*∆*rlmJ* mutant by the number of PFU of the wild type (32). The EOP of the wild type was set to 100. Data are presented as the mean ± standard error of at least three independent experiments. ^***^P < 0.001 (Student’s *t*-test). ns, not significant. The primary receptors are indicated below the diagram.

We screened the most prominent lytic phages for *E. coli,* five of the “T-phages” (27, 29) and the bacteriophage λ (30), for their ability to infect ∆*rlmF*∆*rlmJ* mutant compared to the wild type (Fig. 2). All phages tested are double-stranded DNA phages belonging to the class *Caudoviricetes* and characterized by their tail and icosahedral head (31) (Fig. 2).

Of all lytic phages tested, the T5 phage showed significantly reduced plaque formation in the m^6^A-RNA-depleted ∆*rlmF*∆*rlmJ* mutant compared to the wild type, an effect that was not found with the other phages (Fig. 2).

**Fig 3:**
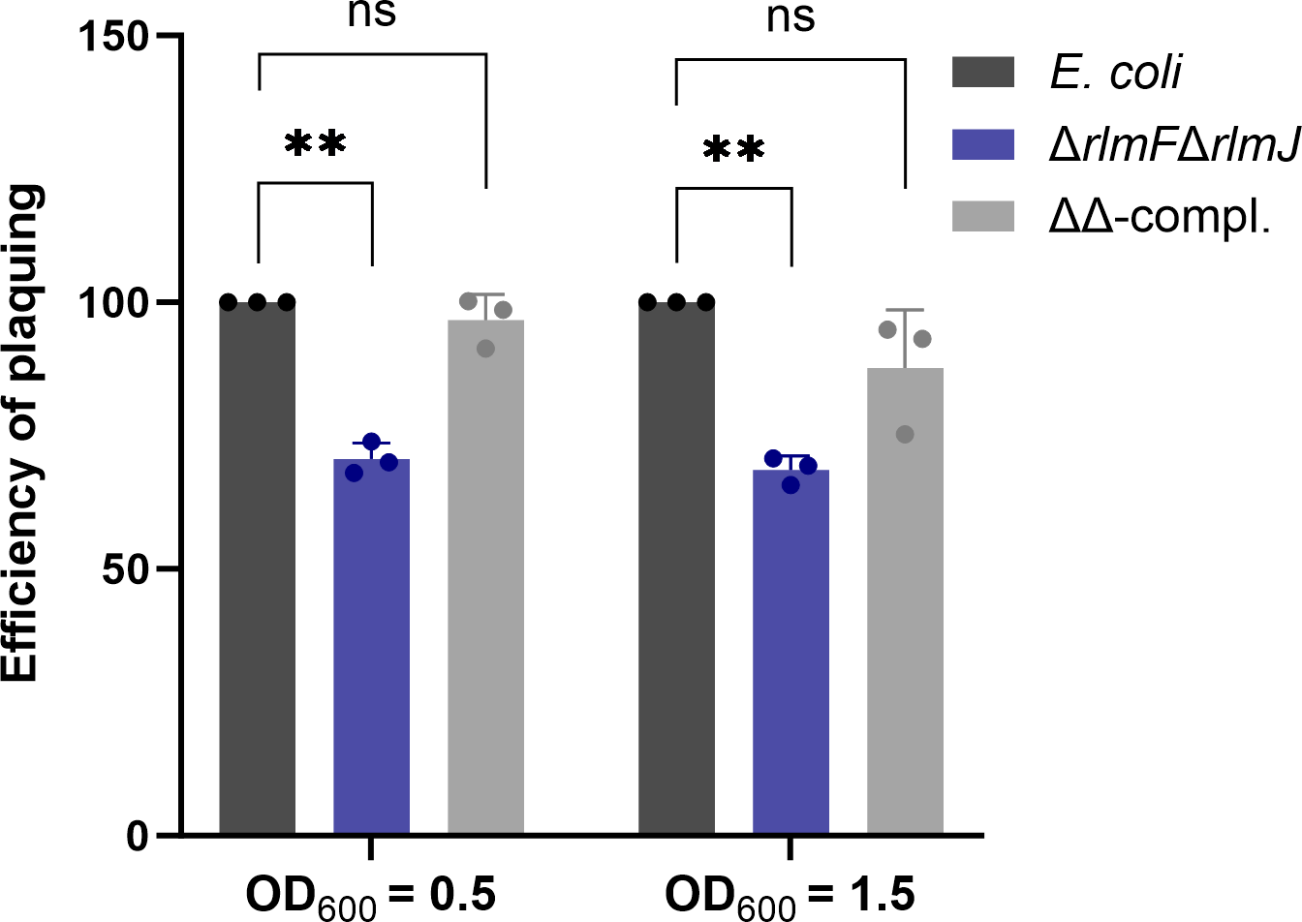
The effect of the growth phase of *E. coli* MG1655 on T5 phage infection. *E. coli* wild type, the ∆*rlmF*∆*rlmJ* mutant, and the complemented mutant (∆∆-compl.) were infected in the exponential growth phase (OD_600_ = 0.5) or stationary phase (OD_600_ = 1.5). The efficiency of plaquing was calculated as in Fig. 2. Data are presented as the mean ± standard deviation of three independent experiments. ^**^P < 0.01 (Student’s *t*-test).

We analyzed the infection by T5 phage at different growth stages of *E. coli* (Fig. 3). The number of plaques was always lower in the ∆*rlmF*∆*rlmJ* mutant compared to the wild type regardless of the growth phase, an effect that was no longer observed in the complemented mutant (∆∆-compl.) (Fig. 3).

To investigate whether the T5 phage possesses its own methyltransferases that could modulate m^6^A-RNA levels during infection, we examined the T5 phage genome for any potential methyltransferase-encoding genes. Since none of the annotated proteins encoded for methyltransferases, we focused on non-annotated T5 proteins in the 250–350 amino acid range, which is typical for most *E*. *coli* methyltransferases. We used AlphaFold 3.0 (33) for structural predictions and compared them in PyMOL (Version 3.0 by Schrödinger) to the known structure of RlmJ and the predicted structure of RlmF. However, no structural similarities were found, suggesting that the T5 phage does not possess its own methyltransferases that could modulate m^6^A. Thus, the lower infection potential of T5 phage is due to the depleted m^6^A-RNA modification in the host.

In a more detailed study, we tested T5-like phages from the BASEL phage collection (34). The BASEL phage collection includes nine newly characterized T5-like phages, all classified under the *Demerecviridae* family within class *Caudoviricetes*. These phages are categorized into two genera according to the International Committee on Taxonomy of Viruses: *Tequintavirus* and *Eseptimavirus*. *Escherichia* phages DaisyDussoix (Bas31), IrisVonRoten (Bas32), HildyBeyeler (Bas33), and SelmaRatti (Bas43) belong to the genus *Tequintavirus*. Like phage T5, these phages belong to the same genus but differ in receptor usage; they use BtuB whereas T5 uses FhuA. In contrast, *Escherichia* phages GreteKellenberger (Bas26), TrudiGerster (Bas27), IrmaTschudi (Bas28), SuperGirl (Bas29), and TrudiRoth (Bas30) are classified under the genus *Eseptimavirus* and also use the BtuB receptor.

**Fig 4:**
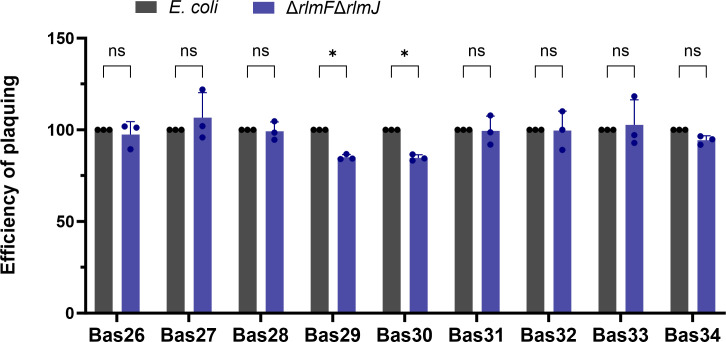
Depletion of m^6^A-RNA in *E. coli* MG1655 and its effect on infection by different T5-like phages. *E. coli* wild type and the ∆*rlmF*∆*rlmJ* mutant were grown to an OD_600_ = 0.5 and infected with different T5-like phages. The EOP was calculated as in Fig. 2. The EOP of the wild type was set to 100. Data are presented as the mean ± standard deviation of three independent experiments. ^*^P < 0.05 (Student’s *t*-test).

Of the nine T5-like phages screened, two phages, SuperGirl (Bas29) and TrudiRoth (Bas30), showed a statistically significant decrease in plaquing efficiency in the m^6^A-RNA-depleted mutant (Fig. 4). These results suggest that the effect of m^6^A-RNA depletion also reduces infection potential in other similar phages but is not a common mechanism.

### m^6^A-RNA depletion increases survival of *E. coli* after bacteriophage T5 infection

**Fig 5:**
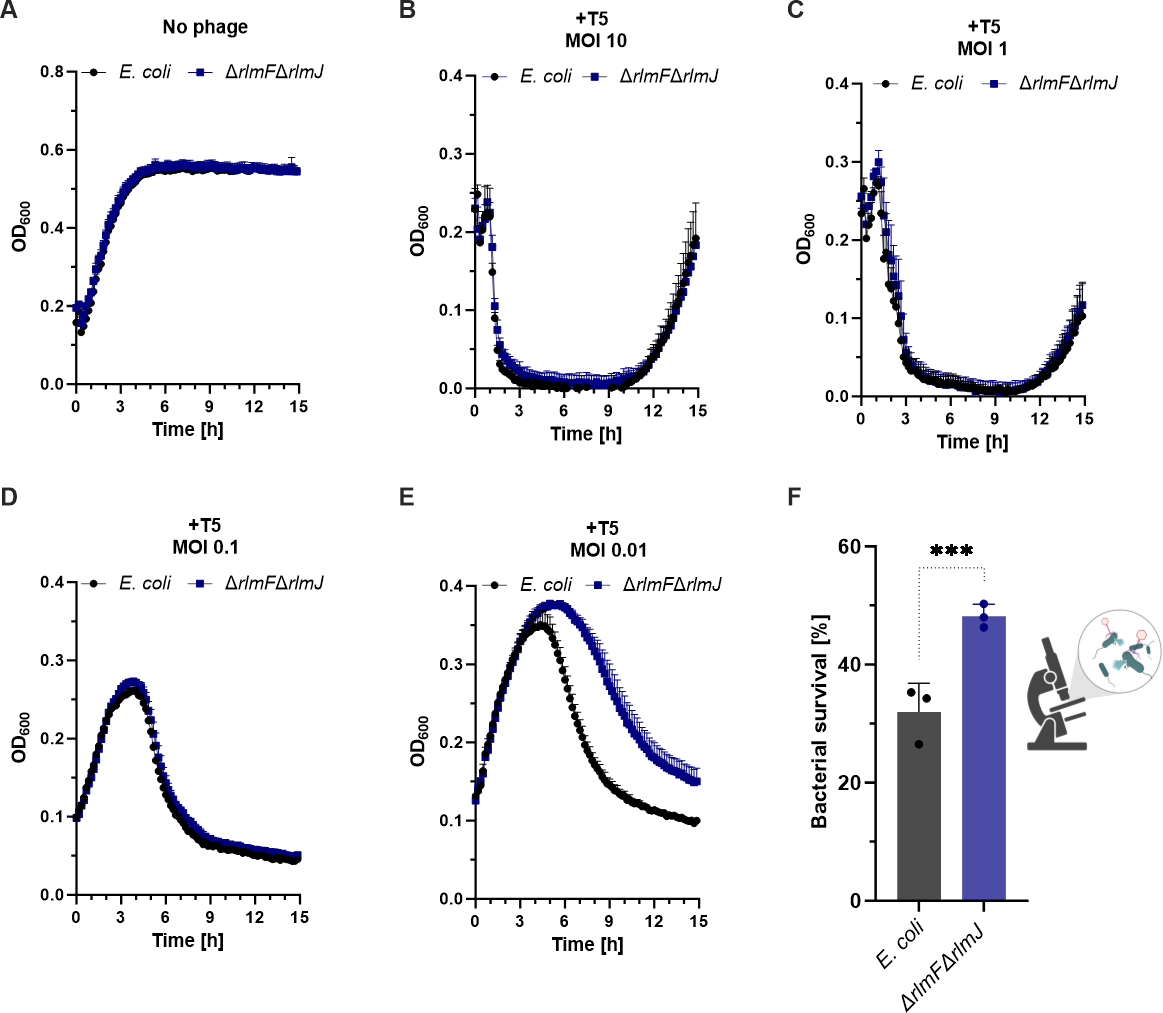
Survival of *E. coli* MG1655 wild type and Δ*rlmF*Δ*rlmJ* mutant after phage T5 infection at different MOIs. (**A–E**) *E. coli* wild type and ∆*rlmF*∆*rlmJ* mutant were grown to the exponential growth phase in nutrient broth. About 2 × 10^8^ cells were non-infected (**A**) or infected with T5 phage at MOI of 10 (**B**), 1 (**C**), 0.1 (**D**), and 0.01 (**E**). The mixtures were transferred to 96-well plates (150 µL per well), and the OD_600_ was measured every 10 min at 37°C with continuous shaking. (**F**) *E. coli* strains were infected with T5 phage at MOI of 10. Single cells were monitored by phase contrast time-lapse microscopy, imaged every 2 min, and the percentage of surviving cells after 2.5 hours was determined. A total of 2,080 wild-type and 1,923 Δ*rlmF*Δ*rlmJ E. coli* cells were quantified at time point 2.5 hours post-infection. Data are presented as the mean ± standard error (**A–D**) or as mean ± standard deviation of the mean (**F**) of three independent biological replicates. ^***^P < 0.001 (Student’s *t*-test).

The reduced efficiency of plaquing of bacteriophage T5 found for the Δ*rlmF*Δ*rlmJ* mutant compared to wild-type *E. coli* (Fig. 2 and 3) prompted us to monitor the survival of both strains at different multiplicities of infection (MOI), which represent the ratio of T5 bacteriophage particles to bacterial cells. In the control experiment (Fig. 5A), bacterial cells proliferated and no lysis was observed. However, the addition of T5 phage resulted in the lysis of bacterial cells (Fig. 5B through E), confirming that the observed phenomenon was a consequence of phage infection. Infection of the exponentially grown *E. coli* wild type and the m^6^A-RNA-depleted mutant with T5 phage at an MOI of 10 or 1 led to the complete collapse of both populations, with earlier collapse at an MOI of 10 (Fig. 5B and C). In both strains, the excess of T5 phage led to rapid cell death. Similarly, infection at an MOI of 0.1 resulted in earlier collapse of both populations than at an MOI of 0.01 (Fig. 5D and E). Strain-specific differences during infection with T5 phage became evident at an MOI of 0.01 (Fig. 5E). The m^6^A-RNA-depleted mutant (Δ*rlmF*Δ*rlmJ*) survived approximately 3 hours longer than the wild type before dramatic cell lysis occurred (Fig. 5E). Furthermore, the cells of the mutant population survived the infection better than the wild type, as about twice as many cells were not lysed (Fig. 5E).

In addition, we analyzed the time-dependent lysis of the bacteria after T5 infection using phase contrast time-lapse microscopy. Exponential phase *E. coli* wild type and Δ*rlmF*Δ*rlmJ* mutant were infected with T5 phage at an MOI of 10 to ensure synchronous phage infection or with nutrient broth as a control. After adsorption for 10 min, samples were spotted on an agarose pad and imaged up to 3 hours post-infection. Images were captured every 2 min in several positions at a constant temperature of 37°C. Intact cells could be easily distinguished from burst cells. A significantly higher proportion of cells from the Δ*rlmF*Δ*rlmJ* population survived after 2.5 hours of T5 infection compared to the *E. coli* wild-type population (Fig. 5F). This result is consistent with the survival experiments (Fig. 5E).

### m^6^A-RNA depletion in *E. coli* does not affect T5 phage absorption but diminishes its infection potential

**Fig 6:**
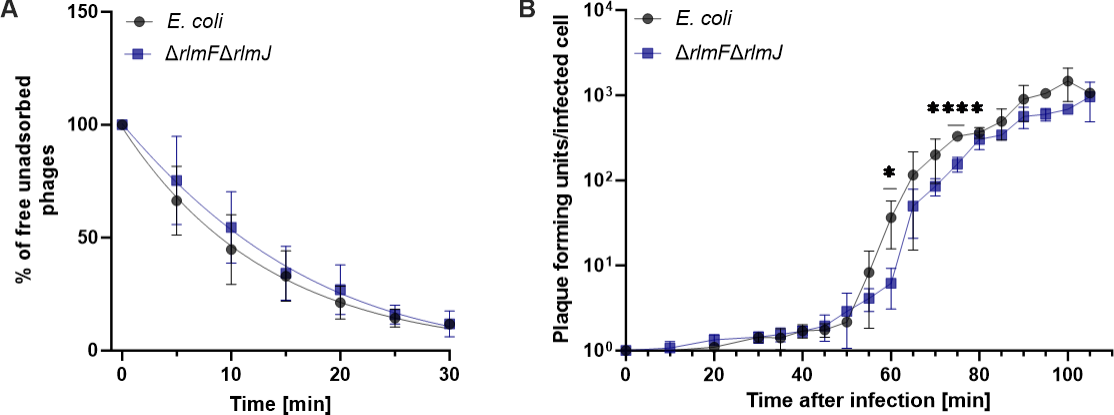
Characterization of T5 phage infection in *E. coli* MG1655 wild type and Δ*rlmF*Δ*rlmJ* mutant. (**A**) Adsorption kinetics of T5 phage to wild type and m^6^A-RNA-depleted mutant *E. coli* at MOI 0.1. Free phages were collected at the indicated time points after infection (0 min), and the concentration of free phages was determined using plaque assays. (**B**) One-step growth curve of T5 phage determined for wild type and m^6^A-RNA-depleted mutant at MOI 0.01. Data are presented as the mean ± standard deviation of at least three independent experiments. ^*^P < 0.05; ^****^P < 0.0001 (paired Student’s *t*-test).

To further investigate T5 phage–host interactions, we investigated the adsorption of T5 to *E. coli* wild type and Δ*rlmF*Δ*rlmJ* mutant (Fig. 6A). We observed similar adsorption rates of T5 phage to both host strains, with at least 60% adsorption within 15 min and 85% adsorption within 25 min (Fig. 6A). These results suggest that the higher percentage of surviving cells observed in the m^6^A-RNA-depleted mutant after T5 phage infection is likely not due to differences in binding to host cells but is instead related to processes that occur after virus–receptor interactions.

To test this, one-step growth curves were performed with the *E. coli* wild type and the Δ*rlmF*Δ*rlmJ* mutant, with the aim of characterizing the dynamics of T5 phage replication. In particular, this analysis allowed us to determine (i) the latent period, the time for the production of phage components, and virion assembly until phage-induced lysis of infected cells, (ii) the rise period, which includes the rise in phage concentration and asynchronous bursts, and (iii) the burst size, a measure of phage virions produced per infected cell (Fig. 6B). For both strains, the latent period was observed to be 45 min, followed by a rise period for another 45 min, followed by a burst period (Fig. 6B). In the Δ*rlmF*Δ*rlmJ* mutant, a significant delay in the increase of the phage titer was observed during the rise period compared to the wild type, which was particularly evident at the 60- and 75-min time points. This period indicates the rise in phage number due to the release of progeny phage particles by lysis of infected bacterial cells. In addition, the burst size for the *E. coli* wild type was 364 phages compared to 339 phages for the Δ*rlmF*Δ*rlmJ* mutant, a reduction of approximately 7% per infection cycle. These results suggest that the m^6^A-RNA-depleted mutant releases progressively fewer phages over multiple rounds of infection, explaining the higher survival rates (Fig. 5E and F) and the lower number of plaque-forming units (PFU) in the mutant (Fig. 3).

## DISCUSSION

In eukaryotes, m^6^A-RNA modifications are crucial for viral infection and host innate antiviral immunity (35). The effect of this modification is highly dependent on the particular virus and cell types involved, leading to either enhancement or inhibition of viral replication in eukaryotes (35).

To date, there are only a few examples of RNA-related modifications known that are important in the context of phage replication (36). ADP-ribosyltransferases (ARTs) of the T4 phage post-translationally modify host proteins with ADP-ribose from the substrate NAD^+^ (37, 38). A recent study reported that one of these enzymes, ModB, accepts not only NAD^+^ but also NAD^+^-capped RNA as a substrate (39). Therefore, RNA chains are covalently attached to host proteins in a process called RNAylation. T4 phages with inactive ModB are characterized by delayed lysis and a reduced number of progenies. The first internal mRNA modification has been described by the group of Michael Laub. They found that the ART CmdT modifies the *N*^6^ position of adenine in GA dinucleotides within single-stranded RNAs leading to an arrest of mRNA translation and inhibition of T4 phage replication (15).

Here, we found that the plaquing efficiency of the T5 and two T5-like phages (Bas29 and Bas30) was significantly reduced in a m^6^A-RNA-depleted *E. coli* mutant compared to the wild type (Fig. 2 to 4). Consequently, more cells of the mutant survived infection with the T5 phage (Fig. 5). Similarly, the lack of the m^6^A reader/writer YTHDF2 and the m^6^A writer METTL3 in eukaryotic BSC40 cell lines attenuates the replication of dsDNA SV40 (11).

Since T5 phage efficiently adsorbed to both the wild type and m^6^A-RNA-depleted mutant of *E. coli* (Fig. 6A), the greater bacterial survival of the mutant likely resulted from changes during the steps after the virus–receptor interaction. The one-step growth curve showed a 5-min delay in the rise period of the m^6^A-RNA-depleted mutant, which was particularly significant at the 60- and 75-min time points (Fig. 6B). This slight delay in the rise period during T5 phage infection may indicate a delay in phage assembly and/or host cell lysis. Strikingly, when analyzing the behavior of single cells, we observed a substantial diversification of the population during T5 phage infection. A larger proportion of m^6^A-RNA-depleted cells survived the infection compared to the wild type (Fig. 5F). We hypothesize that the degree of m^6^A-RNA modification of *E. coli* is heterogeneous and fine-tunes the infection of individual cells by the T5 phage.

In this study, it was shown for the first time that m^6^A-RNA modification of *E. coli* fine-tunes the replication of T5 bacteriophage. Host cells with lower m^6^A-RNA levels have a higher chance of surviving phage infection. We hypothesize that a heterogeneous m^6^A-RNA modification level in individual cells of the *E. coli* population provides a survival advantage because a subset of the population with lower m^6^A-RNA modification levels can escape phage infection and lysis.

## MATERIALS AND METHODS

### Strains, plasmids, and oligonucleotides

The bacterial strains, bacteriophage strains, and plasmids used in this study are listed in Table 1. Oligonucleotide sequences are listed in Table 2.

**TABLE 1:** Bacterial strains, bacteriophages, and plasmids used in this study

Strain	Genotype	Reference
*V. campbellii* ATCC BAA-1116	*V. campbellii* wild type	(22)
*V. campbellii* ∆*rlmJ*	Deletion of *rlmJ* in *V. campbellii*	This work
*V. campbellii* ∆*rlmF*∆*rlmJ*	Deletion of *rlmJ* and *rlmF* in *V. campbellii*	This work
*V. campbellii* ∆*rlmF*∆*rlmJ*+	Insertion of *rlmJ* in *V. campbellii* ∆*rlmF*∆*rlmJ*	This work
*V. campbellii* ∆∆-compl.	Insertion of *rlmF* in the *glmS* region of *V. campbellii* ∆*rlmF*∆*rlmJ+*	This work
*V. harveyi* VH2	GenBank: LGYS00000000	(40)
*E. coli* WM3064	*thrB*1004 *pro thi rpsL hsdS lacZ*ΔM15 RP4-1360 Δ(*araBAD*)567 Δ*dapA*1341::[*erm pir*]	Metcalf, Univ. of Illinois, Urbana
*E. coli* BL21 (DE3)	F^–^*ompT gal dcm lon hsdS_B_* (r*_B_*^–^ m*_B_*^–^) λ(DE3)	(41)
*E. coli* DH5αλpir	*endA1 hsdR17 glnV44* (*= supE44*) *thi-1 recA1 gyrA96 relA1* φ80d*lac*Δ(*lacZ*)M15 Δ(*lacZYA-argF*)U169 *zdg*-232::Tn10 *uidA*::*pir+*	(42)
*E. coli* MG1655	K-12 F^–^ λ^–^ *ilvG^–^ rfb-*50 *rph*-1	(26)
*E. coli* MG1655∆*rlmF*∆*rlmJ*	Deletion of *rlmJ* and *rlmF* in *E. coli*	(28)
*E. coli* MG1655 ∆∆-compl.	Insertion of *rlmF* and *rlmJ* in *E. coli* ∆*rlmF*∆*rlmJ*	(28)
*E. coli* strain B	K-12 F^–^*lon dcm malB*; NCBI Taxonomy ID: 37762; DSM 613	German Collection of Microorganisms and Cell Cultures GmbH (DSMZ)
*E. coli* LE392	*hsd*R514(rk− mk+) *gln*V(supE44) *tryT* (supF58), *lacY1* or Δ(*lacIZY)6, galK2, galT22, metB1, trpR55*; DSM 4230	(43)

Bacteriophage strain	Identifier	Source
Virtus phage	GenBank: OK381870	(25)
T3 phage	DSM 4621; GenBank: NC_047864.1	German Collection of Microorganisms and Cell Cultures GmbH (DSMZ)
T4 phage	DSM 4505; GenBank: AF158101.6	German Collection of Microorganisms and Cell Cultures GmbH (DSMZ)
T5 phage	DSM 16353; GenBank: AY543070.1	German Collection of Microorganisms and Cell Cultures GmbH (DSMZ)
T6 phage	DSM 4622; GenBank: MH550421.1	German Collection of Microorganisms and Cell Cultures GmbH (DSMZ)
T7 phage	DSM 4623; GenBank: NC_001604.1	German Collection of Microorganisms and Cell Cultures GmbH (DSMZ)
λ phage	DSM 4499; GenBank: J02459.1	German Collection of Microorganisms and Cell Cultures GmbH (DSMZ)
vB_EcoS_GreteKellenberger	Bas26	(34)
vB_EcoS_TrudiGerster	Bas27	(34)
vB_EcoS_IrmaTschudi	Bas28	(34)
vB_EcoS_SuperGirl	Bas29	(34)
vB_EcoS_TrudiRoth	Bas30	(34)
vB_EcoS_DaisyDussoix	Bas31	(34)
vB_EcoS_IrisVonRoten	Bas32	(34)
vB_EcoS_HildyBeyerler	Bas33	(34)
vB_EcoS_SelmaRatti	Bas34	(34)

Plasmid	Genotype	Reference
pNTPS138-R6KT	*mobRP4^+^ ori*-R6K *sacB*; suicide plasmid for in-frame deletions, Km^R^	(44)
pNPTS138-R6KT-KO-*rlmF*	pNPTS-138-R6KT-derived suicide plasmid for in-frame deletion of *rlmF* in *V. campbellii,* Km^R^	This work
pNPTS138-R6KT-KO-*rlmJ*	pNPTS-138-R6KT-derived suicide plasmid for in-frame deletion of *rlmJ* in *V. campbellii,* Km^R^	This work
pNPTS138-R6KT-KI-*rlmJ*	pNPTS-138-R6KT-derived suicide plasmid for insertion of *rlmJ* in *V. campbellii* ∆*rlmF*∆*rlmJ,* Km^R^	This work
pNPTS138-R6KT-*glmS*flank-*rlmF-*KI	pNPTS-138-R6KT-derived suicide plasmid for insertion of *rlmF* in the *glmS* region in *V. campbellii* ∆*rlmF*∆*rlmJ*+, Km^R^	This work
pET28a	Expression vector, Km^R^	Novagen
pET28a-V05027	N-terminal His_6_-tagged *VIBHAR_05027, phage major capsid protein* under the control of the T7 promoter, Km^R^	This work
pET28a-V01983	N-terminal His_6_-tagged *VIBHAR_01983, phage major capsid protein* under the control of the T7 promoter, Km^R^	This work

**TABLE 2:** Primers used in this study

Name	Sequence
rlmF_FL_up_fwd	5′-ctggcgccaagcttctctgcaggatgagaaacctaagatgaaagtcttgc-3′
rlmF_FL_OL_rev	5′-ctttaattcaatccaggctttgcgcgcgttgtgattattggtcttcatt-3′
rlmF_FL_OL_fwd	5′-aatgaagaccaataatcacaacgcgcgcaaagcctggattgaattaaag-3′
rlmF_FL_down_rev	5′-gaagctagcgaattcgtggatccagatggctttcagaggcaaaaatcatggc-3′
rlmJ_FL_up_fwd	5′-ctggcgccaagcttctctgcaggatctggtccagcttgggtttgcgttg-3′
rlmJ_FL_OL_rev	5′-tattcaggaacgatccagtcgactttgaagctgtggcggtaacttaacaaa-3′
rlmJ_FL_OL_fwd	5′-tttgttaagttaccgccacagcttcaaagtcgactggatcgttcctgaata-3′
rlmJ_FL_down_rev	5′-agctagcgaattcgtggatccagatgaagaacttggtttcaccagacag-3′
M13_rev	5′-caggaaacagctatgacc-3′
M13_uni	5′-tgtaaaacgacggccagt-3′
rlmJ_check_F	5′-gaccaaatgcggctcaagc-3′
rlmJ_check_R	5′-gctttgacgatggcagcacg-3′
rlmF_check_F	5′-gtgggcatttgtctttgac-3′
rlmF_check_R	5′-caattgaccaatcttggcg-3′
pNPTS_glmS_Fwd	5′- ctggcgccaagcttctctgcaggatgccgcagcaaatcaacgcagcgctg-3′
glmS_rlmF_Rev	5′-gactttcatcttaggtttctcaaaaaagcgaagtcatgtgacttcg-3′
rlmF_glmS_Fwd	5′-gtaattatttagagaaagctatacccgtatatctactgacattgttatcttgttatac-3′
glmS_pNPTS_Rev	5′- gctagcgaattcgtggatccagatcatcaacgaaaccgagatcattgag-3′
glmS_rlmF_Fwd	5′-cgaagtcacatgacttcgcttttttgagaaacctaagatgaaagtc-3′
rlmF_glmS_Rev	5′-gtataacaagataacaatgtcagtagatatacgggtatagctttctctaaataattac-3′
VC_glmS_check_F	5′-gccttcacgactcagctctc-3′
VC_glmS_check_R	5′-cgagacactttacaaccaac-3′
V05027-EcoRI-fwd	5′-tagccgaattcatgcttaatgccgtatcgactc-3′
V05027-Xhol-rev	5′-tagccctcgagttaccccttcgcagattcaacaacg-3′
V01983-EcoRI-fwd	5′-tagccgaattcatgcctgataattacaccactcg-3′
V01983-Xhol-rev	5′-tagccctcgagttaaccgactttgacgacaacaa-3′
pET28a_check_F	5′-aagtggcgagcccgatcttcc-3′
pET28a_check_R	5′-gatatagttcctcctttcagc-′

### Plasmid and bacterial strain construction

Molecular methods were carried out according to the manufacturer’s instructions. Chromosomal DNA isolation, plasmid isolation, and PCR product clean-up kits were purchased from Süd-Laborbedarf Gauting (Gauting, Germany). Enzymes and HiFi DNA Assembly Master Mix were purchased from New England BioLabs (Ipswich, MA, USA).

Construction of the marker-less double deletion mutant *V. campbellii* ∆*rlmF*∆*rlmJ* was achieved using the suicide plasmids pNPTS138-R6KT-KO-*rlmJ* and pNPTS138-R6KT-KO-*rlmF*. Briefly, 600-bp regions upstream and downstream of *rlmJ* or *rlmF* were amplified by PCR using genomic DNA from *V. campbellii* as a template. The primers rlmJ_FL_up_fwd and rlmJ_FL_OL_rev were used for the upstream fragment of *rlmJ*, rlmJ_FL_OL_fwd and rlmJ_FL_down_rev rev for the downstream fragment of *rlmJ*, rlmF_FL_up_fwd and rlmF_FL_OL_rev for the upstream fragment of *rlmF*, and rlmF_FL_OL_fwd and rlmF_FL_down_rev for the downstream fragment of *rlmF*. After purification, the PCR fragments were cloned by Gibson assembly (45) into the suicide plasmid pNPTS138-R6KT, which was first linearized by digestion with EcoRV. The correct plasmids were verified by colony PCR and sequencing using primers M13_rev and M13_uni.

The suicide plasmid pNPTS138-R6KT-KO-*rlmJ* was next introduced into wild-type *V. campbellii* by conjugative mating using *E. coli* WM3064 as a donor in the LB medium containing diaminopimelic acid (DAP) as previously described (46). Briefly, single-crossover integration mutants were selected on LM plates that contained kanamycin but lacked DAP. Single colonies were grown for 2 days without antibiotics and plated on LB containing 10% (wt/vol) sucrose to select for plasmid excision. Kanamycin-sensitive colonies were checked for gene deletion by colony PCR using primers bracketing the deletion sites. The deletion of *rlmJ* was verified by colony PCR and sequencing using the primers rlmJ_check_F and rlmJ_check_R. *rlmF* was then deleted through the introduction of the suicide plasmid pNPTS138-R6KT-KO-*rlmF* into *V. campbellii* ∆*rlmJ* via conjugative mating using *E. coli* WM3064 as a donor as described above. Deletion of *rlmF* was verified by colony PCR and sequencing using the primers rlmF_check_F and rlmF_check_R.

Complementation of the *rlmJ* deletion in *V. campbellii* ∆*rlmF*∆*rlmJ* was achieved with the suicide plasmid pNPTS138-R6KT-KI*-rlmJ*. Briefly, *rlmJ* and 600 bp upstream and downstream flanking regions were amplified by PCR using genomic DNA from *V. campbellii* as a template and the primers rlmJ_FL_up_fwd and rlmJ_FL_down_rev. After purification, the 2,030-bp-long PCR fragment was cloned by Gibson assembly (45) into the suicide plasmid pNPTS138-R6KT, which was linearized by digestion with EcoRV. The correct plasmids were verified by colony PCR and sequencing using primers M13_rev and M13_uni. Construction of the suicide plasmid pNPTS138-R6KT-*glmS*flank-*rlmF-*KI was slightly different due to the presence of transposable elements in the vicinity of the gene. Briefly, the regions surrounding *glmS* and *rlmF* (600 bp upstream and 24 bp downstream) were amplified to generate the suicide plasmid pNPTS138-R6KT-*glmS*flank-*rlmF* using *V. campbellii* genomic DNA as a template. Primer pairs used for amplification of *glmS* overhang fragments were pNPTS_glmS_Fwd and glmS_rlmF_Rev (upstream of *rlmF*) and rlmF_glmS_Fwd and glmS_pNPTS_Rev (downstream from *rlmF*). The primers glmS_rlmF_Fwd and rlmF_glmS_Rev were used to amplify *rlmF*. These DNA fragments were assembled via Gibson assembly (45) into the pNPTS138-R6KT plasmid linearized with EcoRV. The plasmid was verified by colony PCR and sequencing using primers M13_rev and M13_uni.

To complement ∆*rlmF*∆*rlmJ*, the suicide plasmid pNPTS138-R6KT-KI*-rlmJ* was introduced into *V. campbellii* ∆*rlmF*∆*rlmJ* by conjugative mating using *E. coli* WM3064 as a donor in the LB medium containing DAP as described above. Insertion of *rlmJ* was verified by colony PCR and sequencing using the primers rlmJ_check_F and rlmJ_check_R. *rlmF* was then integrated via the introduction of suicide plasmid pNPTS138-R6KT-*glmS*flank-*rlmF-*KI in *V. campbellii* ∆*rlmF*∆*rlmJ+* by conjugative mating with *E. coli* WM3064 as described above. Insertion of *rlmF* in the *glmS* region of *V. campbellii* was verified by colony PCR and sequencing using the primers VC_glmS_check_F and VC_glms_check_R.

To generate plasmids encoding N-terminal 6His-tagged versions of major capsid proteins from *Vibrio* kappa-like prophage and ΦHAP-1-like prophage (*VIBHAR_05027* and *VIBHAR_01983,* respectively), *V. campbellii* genomic DNA was used as a template with primers V05027-EcoRI-fwd and V05027-Xhol-rev (*VIBHAR_05027*) or V01983-EcoRI-fwd and V01983-Xhol-rev (*VIBHAR_01983*). These two genes were cloned into the pET-28a vector using EcoRI and XhoI as restriction sites; the presence of the correct insert was confirmed by colony PCR and sequencing using primers pET28a_check_F and pET28a_check_R.

### Mass spectrometry analysis of mRNA and rRNA methylation in *V. campbellii*

To identify m^6^A methylation of the rRNA and mRNA moieties in wild type and ∆*rlmF*∆*rlmJ* mutant *V. campbellii*, total RNA was isolated and processed as previously described (16). In brief, *V. campbellii* cells were grown to an OD_600_ of 1 and harvested by centrifugation. Total RNA was isolated, and tRNA was removed by size exclusion chromatography, resulting in a fraction containing mRNA and rRNA moieties. This RNA fraction was hydrolyzed and quantitatively analyzed by LC-MS/MS (16).

### Overproduction and purification of recombinant phage capsid proteins

*E. coli* BL21 (DE3) was transformed with the vectors pET28a-V05027 and pET28a-V01983 to overexpress the major capsid proteins of *Vibrio* kappa-like prophage and ΦHAP-1-like prophage, respectively. Cells were cultivated in LB supplemented with kanamycin (50 mg/mL) at 37°C to an OD_600_ of 0.5. Isopropyl-β-D-1-thiogalactopyranoside (0.5 mM) was added to the culture to induce *VIBHAR_05027* or *VIBHAR_01983* expression at 37°C for 2.5 hours. Cells were harvested (5,000 × *g*, 20 min, 4°C), resuspended, and disrupted in ice-cold disruption buffer [50 mM Tris-HCl (pH 7.5), 10% (vol/vol) glycerol, 10 mM magnesium chloride, 1 mM dithiothreitol, 0.5 mM phenazine methosulfate, 3 mg DNase, and 100 mM NaCl] using a high-pressure cell disrupter (Constant Systems Limited, Daventry, UK). Cell debris and intact cells were removed by centrifugation (5,000 × *g*, 20 min, 4°C), and the pellet was solubilized in 6 M urea and 10 mM Tris-HCl (pH 7.5) overnight with shaking at 4°C. Any precipitates or unsolubilized aggregates were removed by ultracentrifugation (20,000 × *g*, 15 min, 4°C), and the supernatant was loaded onto a Ni-nitrilotriacetic acid column (Qiagen, Hilden, Germany). After a washing step [6 M urea, 10 mM Tris-HCl (pH 7.5), and 40 mM imidazole], the recombinant protein was eluted with elution buffer [6 M urea, 10 mM Tris-HCl (pH 7.5), and 200 mM imidazole].

### Generation of polyclonal rabbit antibodies against recombinant phage capsid proteins

Customized polyclonal rabbit antibodies against 6His-VIBHAR_05027 and 6His-VIBHAR_01983 were purchased from Kaneka Eurogentec (Seraing, Belgium). Heterologously produced and purified 6His-VIBHAR_05027 or 6His-VIBHAR_01983 were used as antigens in a Speedy 28-day Immunization Program with two rabbits per antigen as hosts. The specificity of the polyclonal antibodies against 6His-VIBHAR_05027 and 6His-VIBHAR_01983 was verified by western blot analysis as described below.

### Sodium dodecyl sulfate-polyacrylamide gel electrophoresis and western blot analysis of 6His-VIBHAR_05027 and 6His-VIBHAR_01983

#### Sodium dodecyl sulfate-polyacrylamide gel electrophoresis

Stacking gels consisted of 4% (wt/vol) acrylamide [in 50 mM Tris (pH 6.8)] and resolving gels of 12.5% (wt/vol) acrylamide [in 300 mM Tris (pH 8.8)] and were run in a Tris-glycine buffer [25 mM Tris, 192 mM glycine, 0.1% (wt/vol) SDS (pH 8.3) (47)]. Gels were stained overnight in Coomassie staining solution [0.25% (wt/vol) Coomassie Brilliant Blue R-250, 9.2% (vol/vol) concentrated acetic acid and 45.4% (vol/vol) ethanol] and destained in destaining solution [10% (vol/vol) acetic acid and 40% (vol/vol) ethanol].

#### Western blot analysis

Proteins were transferred to a nitrocellulose membrane using the Trans-Blot Turbo Transfer System (Bio-Rad, Hercules, CA, USA) and then blocked with 5% (wt/vol) milk powder prepared in Tris-buffered saline (TBS) (pH 7.6) containing 0.1% (vol/vol) Tween-20 (TBST) for 1 hour at room temperature. 6His-VIBHAR_05027 and 6His-VIBHAR_01983 were detected by incubating the membrane in TBST with primary polyclonal antibodies (Kaneka Eurogentec, Seraing, Belgium) or primary monoclonal antibodies against the 6His-tag (Thermo Fisher Scientific, Waltham, MA, USA). Membranes were then incubated with the alkaline phosphatase-conjugated goat anti-rabbit IgG (Thermo Fisher Scientific, Waltham, MA, USA) and developed using substrate solution [50 mM sodium carbonate buffer (pH 9.5), 0.1% (wt/vol) nitroblue tetrazolium, and 5 mg/mL 5-bromo-4-chloro-3-indolyl phosphate]. PageRuler Prestained Protein ladder (10–180 kDa) (Thermo Fisher Scientific, Waltham, MA, USA) was used as a size standard ladder.

### Phage induction assays

Wild type, ∆*rlmF*∆*rlmJ* mutant, and complemented mutant (∆∆-compl.) *V. campbellii* were exposed to stress conditions to assess the effect of m^6^A-RNA modification on prophage induction. Bacterial strains grown in the LM medium to an OD_600_ of 0.4 were exposed to either oxidative stress (by the addition of 1 µg/mL mitomycin C), heat stress (45°C), or neither (control). After 30 min of oxidative stress, mitomycin C was removed from the sample by centrifugation (5,000 × *g*, 10 min, 4°C), and the pellet was resuspended in fresh, prewarmed LM medium. After 30 min of heat stress, the samples were shifted to 30°C. After 2 hours of incubation under physiological conditions, the supernatant containing the phage particles was collected (5,000 × *g*, 15 min, 4°C), passed through a 0.22 µm Millipore Steriflip Vacuum filter (MilliporeSigma, Burlington, MA, USA), and stored at 4°C.

To follow cell lysis caused by the release of lysogenic phages, wild type, ∆*rlmF*∆*rlmJ* mutant, and complemented mutant (∆∆-compl.) *V. campbellii* were exposed to 1 µg/mL mitomycin C after strains were grown in the LM medium to an OD_600_ of 0.4. After 30 min of oxidative stress, mitomycin C was removed from the sample by centrifugation (5,000 × *g*, 10 min, 4°C), and the pellet was resuspended in fresh, prewarmed LM medium. The mixtures were transferred to 96-well plates (150 µL per well), and OD_600_ was measured every 10 min using a NanoQuant Infinite M200PRO plate reader (Tecan, Männedorf, Switzerland) at 37°C with continuous shaking.

### Indirect enzyme-linked immunosorbent assay

Indirect ELISA was performed according to the described protocols (48, 49) with slight modifications for the detection of the major capsid proteins of *Vibrio* kappa-like and ΦHAP-1-like prophages from *V. campbellii*. In brief, 96-well F-bottom, polystyrene, chimney well, black, medium binding microplates (Greiner Bio-One GmbH, Kremsmünster, Austria) were coated with 100 µL of supernatant (derived from the phage induction assays described above) and 0.05 M carbonate buffer (pH 9.6) and then incubated overnight at 4°C. After washing three times with TBST, each well of the plate was blocked with 200 µL of 5% (wt/vol) milk powder prepared in TBST for 1 hour at 30°C. After washing three times with TBST, 100 µL of VIBHAR_05027- or VIBHAR_01983-positive serum in 5% (wt/vol) milk powder with TBST was added to each well, and the plate was incubated for 1 hour at 30°C. After three washes with TBST, 100 µL of alkaline phosphatase-conjugated goat anti-rabbit IgG (Thermo Fisher Scientific, Waltham, MA, USA) diluted in 5% (wt/vol) milk powder in TBST was added to each well, following incubation at 30°C for 1 hour and five washes with TBST. Subsequently, 100 µL of the substrate para-nitrophenyl phosphate (Thermo Fisher Scientific, Waltham, MA, USA) was added to each well and incubated for 30 min in the dark. Last, 50 µL of 2 N sodium hydroxide was added to each well to stop the reaction. The absorbance was immediately measured at 405 nm in a NanoQuant Infinite M200PRO plate reader (Tecan, Männedorf, Switzerland). All samples were analyzed in technical duplicates.

Standard curves generated with purified major capsid proteins were included in each ELISA run to estimate the concentration of the two major capsid proteins in the sample.

### Propagation and determination of phage titer using plaque assay

To obtain high-titer phage stocks, propagation of all phages mentioned in this work was performed using the plate lysate method as described (50). The propagation host used for Virtus phage was *V. harveyi* VH2; the λ phage host was *E. coli* DSM 4230; and the host for all T phages was *E. coli* B strain (DSM 613).

To determine the phage titer, a double agar overlay plaque assay was performed as described (51). In brief, 100 µL of exponential-phase bacterial culture was mixed with 100 µL of diluted phages in soft agar, which was then poured onto a solid medium. The plates were incubated overnight, and the number of plaques formed was counted to determine the titer.

### Efficiency of plaquing

EOP was determined after performing the plaque assay described above, using phage lysates to infect wild type and ∆*rlmF*∆*rlmJ* mutants of *V. campbellii* and *E. coli*. The EOP was calculated by dividing the number of PFU of the ∆*rlmF*∆*rlmJ* mutant by the number of PFU of the wild type (32). The EOP of the wild type was set to 100.

### Bacterial survival after T5 phage infection

*E. coli* wild type and ∆*rlmF*∆*rlmJ* mutant were grown to the exponential growth phase in nutrient broth (N7519, Sigma-Aldrich, St. Louis, MO, USA). Cells (about 2 × 10^8^ CFU/mL) were infected with T5 phage at a MOI of 10, 1, 0.1, or 0.01 to monitor host dynamics. Mixtures were transferred to 96-well plates (150 µL per well), and OD_600_ was measured every 10 min using a NanoQuant Infinite M200PRO plate reader (Tecan, Männedorf, Switzerland) at 37°C with continuous shaking.

### Synchronized infection assay

The synchronized infection assay was performed as previously described (52, 53). *E. coli* wild type and ∆*rlmF*∆*rlmJ* mutant were grown as described above and infected with T5 phage at a MOI of 10. Samples were collected at 0 and 5 min after infection and immediately plated for CFU enumeration. Three biologically independent infection experiments showing a CFU reduction of at least 95% within 5 min of infection were considered as synchronized infection.

### Time-lapse phase contrast microscopy

Phase contrast time-lapse microscopy was used to follow T5 phage-mediated bacterial lysis using a modified protocol described by Mandal and colleagues (54). *E. coli* wild type and ∆*rlmF*∆*rlmJ* mutant were grown as described above and infected with T5 phage at a MOI of 10 (or with an equal volume of phage diluent as a control). After a 10-min incubation for initial adsorption at 37°C under agitation, 2 µL of cells was spotted on pads composed of nutrient broth (N7519, Sigma-Aldrich, St. Louis, MO, USA) and 1% (wt/vol) agarose. These pads were placed on microscopic slides and covered with a coverslip. Within 20 min, images were taken on a Leica DMi8 inverted microscope equipped with a Leica DFC365 FX camera (Wetzlar, Germany). Microscopic phase contrast images were captured every 2 min in several positions up to 3 hours with a constant temperature of 37°C using an incubator (PeCon, Erbach, Germany) around the DMi8 microscope. To set the focus plane, automatic autofocus was performed at every position and every time point using the adaptive focus control and closed-loop focus system of the DMi8 microscope. To quantify non-lysed cells, phase contrast images were analyzed using the plugin MicrobeJ (55) in ImageJ (56). The default MicrobeJ settings were used for cell segmentation (Fit shape, rod-shaped bacteria) apart from the following settings: area: 1-max µm; length: 2.5–20 µm; width: 0.4–2 µm; curvature: 0.0–0.15; angularity: 0.0–0.25; and intensity: 0.0–2,000.

### T5 bacteriophage adsorption assay

Adsorption assays were performed as previously described (57). *E. coli* wild type and ∆*rlmF*∆*rlmJ* mutant were grown as described above and infected with T5 phage at an MOI of 0.1. Free phages were sampled immediately post-infection (0 min), then every 5 min for 30 min using 0.22 µm sterile filtration. Plaque assays (51) were performed as described above to enumerate the free phage concentration.

### One-step growth curve

One-step growth curves were performed as described previously (52, 58). *E. coli* wild type and ∆*rlmF*∆*rlmJ* mutant were grown as described above, infected with T5 phage at an MOI of 0.001, and incubated at 37°C for 10 min. After initial adsorption, the remaining free phages were removed via centrifugation (5,000 × *g*, 5 min, 4°C), and the pellet was resuspended in fresh prewarmed nutrient broth. Samples were incubated at 37°C with shaking and collected (including *t* = 0) at 10-min intervals over 30 min, followed by 5-min intervals up to 105 min. Plaque assays (51) were performed as described above immediately after sample collection.

### Statistical analysis and data presentation

All numerical data were analyzed, and graphs were prepared with GraphPad Prism version 10.0.2 (GraphPad Software, La Jolla, CA, USA). Statistics were performed using paired and unpaired Student’s *t*-test. Microscopy images were prepared from a total of 2,080 *E. coli* wild type and 1,923 ∆*rlmF*∆*rlmJ* mutant cells and analyzed with MicrobeJ. All figures were generated using Affinity Designer version 2.2.1 (Serif, West Bridgford, UK).

All experiments were repeated at least three times to ensure reproducibility.

## Data Availability

Additional information required to reanalyze the data reported in this paper is available from the lead contacts upon request.
